# lncRNA Signature for Predicting Cerebral Vasospasm in Patients with SAH: Implications for Precision Neurosurgery

**DOI:** 10.1016/j.omtn.2020.07.028

**Published:** 2020-07-25

**Authors:** Chen-Yu Pan, Miao Tian, Lei-Lei Zhang, Dan Tian, Li-Yan Wang, Yu-Jia Sun, Yun-Feng Cui

**Affiliations:** 1 Department of Anesthesiology, The Second Hospital of Jilin University, Changchun, China; 2 Department of Gynecology and Obstetrics, The Second Hospital of Jilin University, Changchun, China; 3 Department of Operating Room, The Second Hospital of Jilin University, Changchun, China

**Keywords:** subarachnoid hemorrhage, cerebral vasospasm, long non-coding RNA, MALAT1, LINC01619

## Abstract

Subarachnoid hemorrhage (SAH) patients’ surgery is performed to prevent extravasation of blood into the subarachnoid space. Cerebral vasospasm (CVS; narrowing of cerebral arteries) occurs following SAH and represents a major cause of associated mortality and morbidity. To improve postsurgery care of SAH patients and their prognosis, the ability to predict CVS onset is critical. We report a long noncoding RNA (lncRNA) signature to distinguish SAH patients with CVS from SAH patients without CVS. Cerebrospinal fluid (CSF) was obtained from SAH patients without CVS (n = 10) and SAH patients with CVS (n = 10). lncRNAs ZFAS1 and MALAT1 were significantly upregulated (p < 0.05), whereas lncRNAs LINC00261 and LINC01619 were significantly downregulated in SAH patients with CVS (p < 0.05) compared to SAH patients without CVS. We applied this lncRNA signature to retrospectively predict CVS in SAH patients (n = 38 for SAH patients without CVS, and n = 27 for SAH patients with CVS). The 4-lncRNA signature was found to be predictive in >40% of samples and the 2-lncRNA comprising MALAT1 and LINC01619 accurately predicted CVS in ∼90% cases. These results are initial steps toward personalized management of SAH patients in clinics and provide novel CSF biomarkers that can substantially improve the clinical management of SAH patients.

## Graphical Abstract

**Figure:**
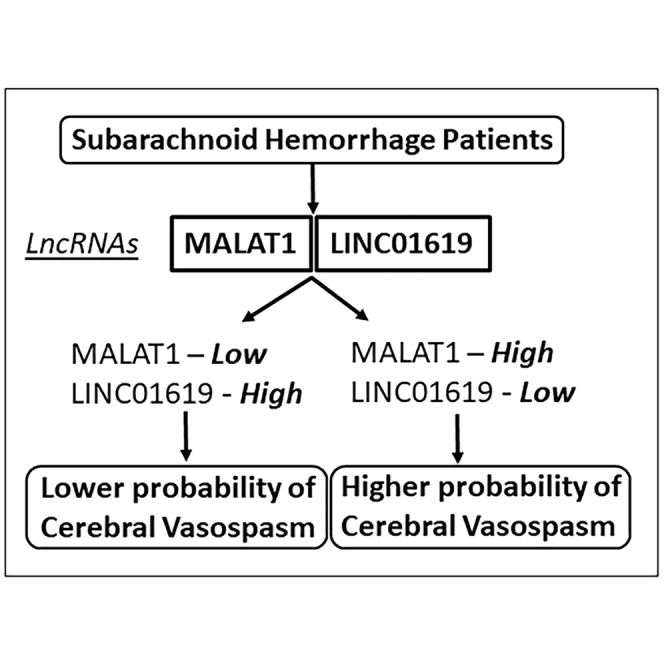

## Teaser

Cerebral vasospasm (CVS) is a major cause of subarachnoid hemorrhage (SAH) mortality and morbidity. However, the prediction of CVS remains a clinical challenge. Yun-Feng Cui and colleagues report that lncRNAs MALAT1 and LINC01619 can predict CVS occurrence with remarkable precision. This work is a step toward personalized treatment and management of SAH patients.

## Introduction

Subarachnoid hemorrhage (SAH) is a condition that is marked by bleeding into a subarachnoid space, the area surrounding brain in between the arachnoid membrane and the pia mater.^1^^,^^2^ It is a condition with a high rate of fatality and permanent disability.^3^ The spontaneous occurrence of SAH is about one in 10,000 individuals per year, and the risk includes factors, such as high blood pressure, family history, smoking, etc.^1^ A population study in China recruited 512,891 adults (41% men and 59% women) to study the occurrence and risk factors for SAH.^4^ It found an annual incidence rate of 12.9 per 100,000 individuals or 1.29 per 10,000 individuals. Thus, the incidence of SAH in China is quite comparable to the numbers in Western countries. The Chinese study also found a connection between higher blood pressure and SAH—an average of 10 mmHg higher systolic blood pressure and an average of 5 mmHg higher diastolic blood pressure in individuals suffering with SAH.^4^

The knowledge on etiology of SAH remains inadequate, and not much is known about its pathogenesis.^4^ Other than trauma, intracranial aneurysm is the most common cause of SAH, and it is estimated that 15%–30% of patients with aneurysmal SAH die before ever reaching a hospital. 25% patients die within the first 24 h, and the mortality at the end of the first week is approximately 40%. Cerebral vasospasm (CVS) is a well-known phenomenon that happens subsequent to aneurysmal SAH. It is the narrowing of intracranial arteries,^5^ and CVS leading to delayed cranial ischemia is a major complication and source of morbidity in patients with aneurysmal SAH.^6^

Recent years have witnessed a spurt in investigations focused on noncoding RNAs as diagnostic or prognostic markers.^7^^,^^8^ Stylli et al.^9^, for example, sought a miRNA (microRNA) signature for patients with SAH, who develop CVS, compared to those who don’t. They identified microRNA (miR)-451a and miR-27a-3p as the two miRNAs that were differentially expressed in SAH with CVS patients compared to SAH patients who never had CVS. This was an interesting work, documenting, for the first time, a miRNA signature that can enormously help neurosurgeons. However, additionally, the long noncoding RNAs (lncRNAs) have never been evaluated for such possible signature, even though they have been shown to be mechanistically involved in several neurological disorders.^10^^,^^11^

## Results

### Differentially Expressed lncRNAs in Pooled Samples

**Figure 1:**
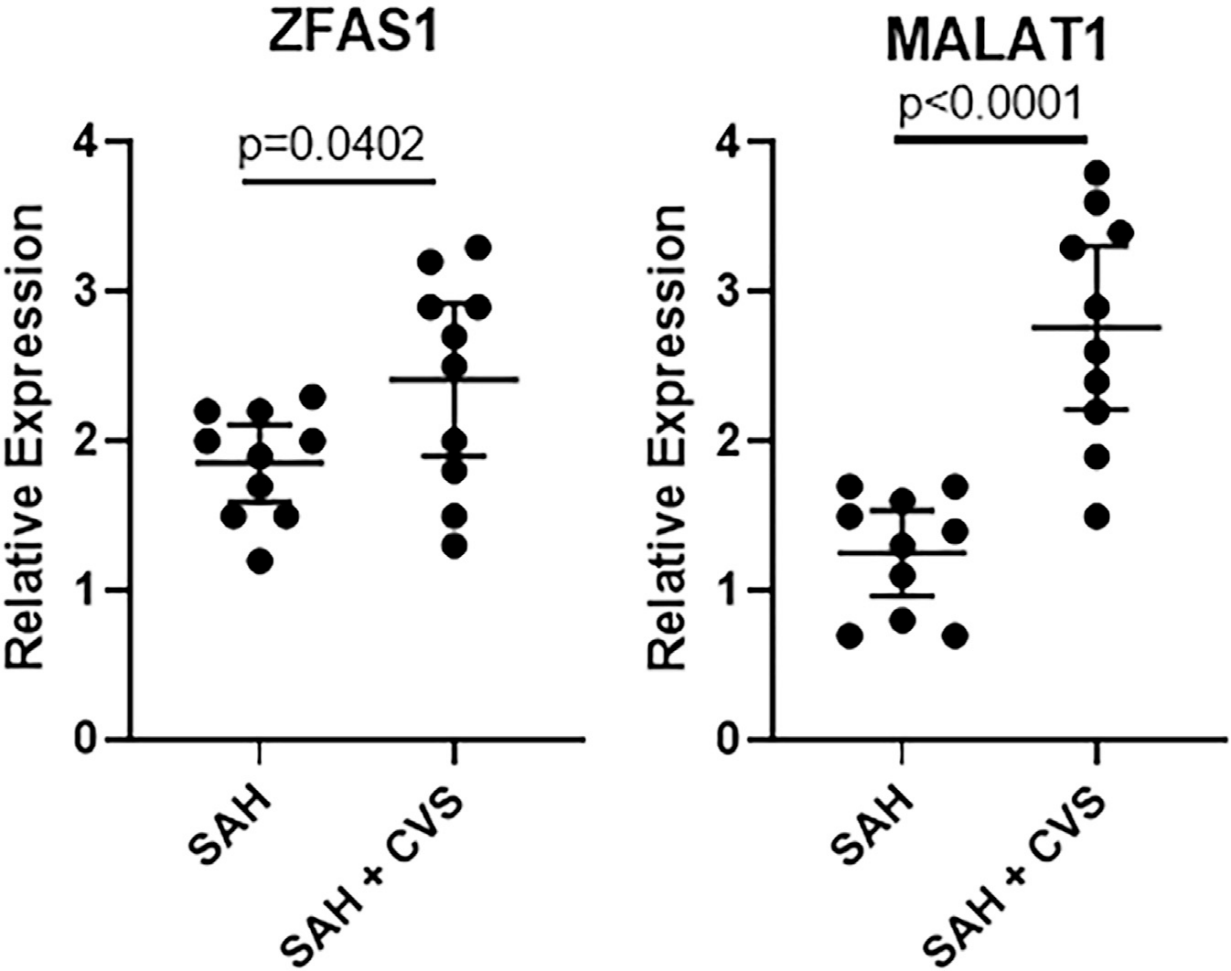
lncRNAs ZFAS1 and MALAT1 in SAH Patients Expression levels of lncRNAs ZFAS1 and MALAT1 in pooled samples representing patients with SAH (without CVS) and those with SAH and CVS. Values plotted are mean ± 95% confidence interval (CI).

We screened for differentially expressed lncRNAs in patient samples to be able to predict CVS in SAH patients. First, the screening for lncRNAs was conducted in pooled samples. A total of 20 lncRNAs were screened, based on the information from “LNCipedia” and “NONCODE.” RNA samples from patients representing a single group were pooled together for an initial screening. The two lncRNAs that were upregulated in SAH patients with CVS, compared to SAH-alone patients, were ZFAS1 and MALAT1 (Figure 1). For ZFAS, the mean expression for SAH patients without CVS was 1.85 with 0.36 SD, whereas that for SAH patients with CVS was 2.41 with 0.71 SD, with the p value being 0.0402 between the groups (p < 0.05). For MALAT1, the mean expression for SAH patients without CVS was 1.25 with 0.40 SD, whereas that for SAH patients with CVS was 2.76 with 0.77 SD, and the statistical difference between the groups was highly significant (p < 0.0001) (Figure 1).

**Figure 2:**
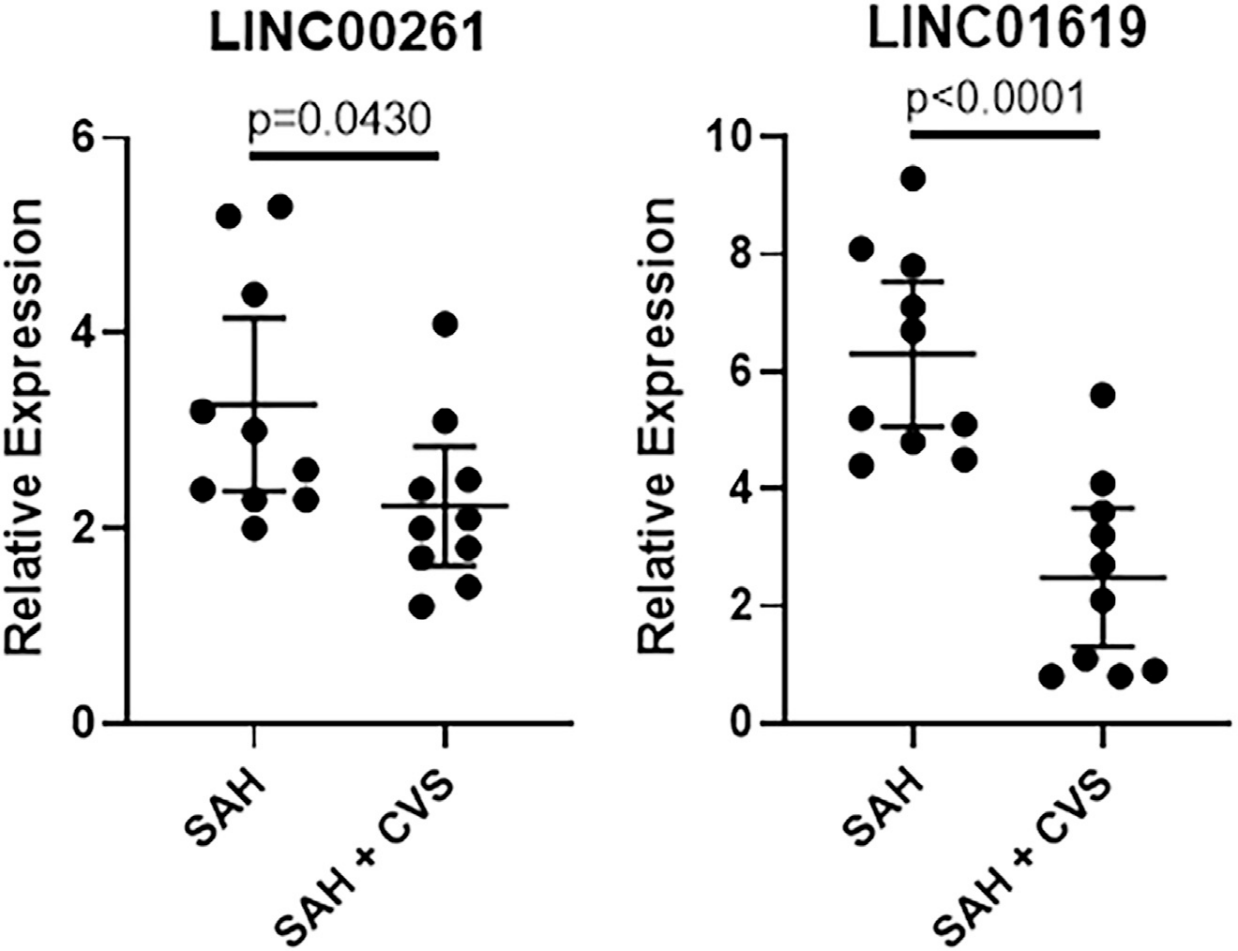
lncRNAs LINC00261 and LINC01619 in SAH Patients Expression levels of lncRNAs LINC00261 and LINC01619 in pooled samples representing patients with SAH (without CVS) and those with SAH and CVS. Values plotted are mean ± 95% CI.

The two most significantly downregulated lncRNAs were LINC00261 and LINC01619 (Figure 2). For LINC00261, the mean expression for SAH patients without CVS was 3.27 with 1.24 SD, whereas that for SAH patients with CVS was 2.23 with 0.86 SD (p = 0.0430). For LINC01619, the mean expression for SAH patients without CVS was 6.3 with 1.73 SD, whereas that for SAH patients with CVS was 2.49 with 1.65 SD. The difference between the groups was highly significant (p < 0.0001) (Figure 2).

### Expression of ZFAS1, MALAT1, LINC00261, and LINC01619 lncRNAs in Individual Samples

The analyses of pooled samples suggested the possible power of four lncRNAs to help distinguish SAH patients who will develop CVS from those who won’t. However, we realize that many times, a few individual samples with aberrant expression can influence the overall outcome. Therefore, we next evaluated the expression levels of these four lncRNAs in all of the individual samples.

**Figure 3:**
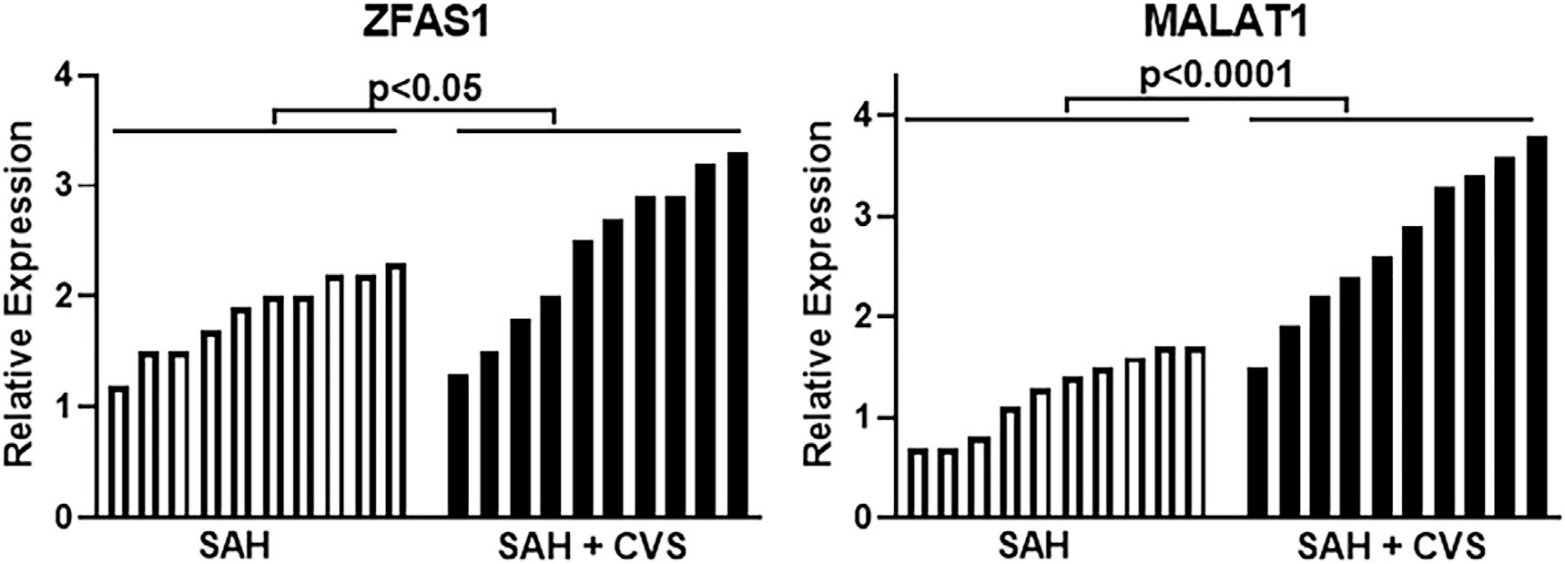
Expression of ZFAS1 and MALAT1 in Individual Patient Samples Expression levels of lncRNAs ZFAS1 and MALAT1 in individual samples representing patients with SAH (without CVS) and those with SAH and CVS. Values plotted are mean ± 95% CI.

**Figure 4:**
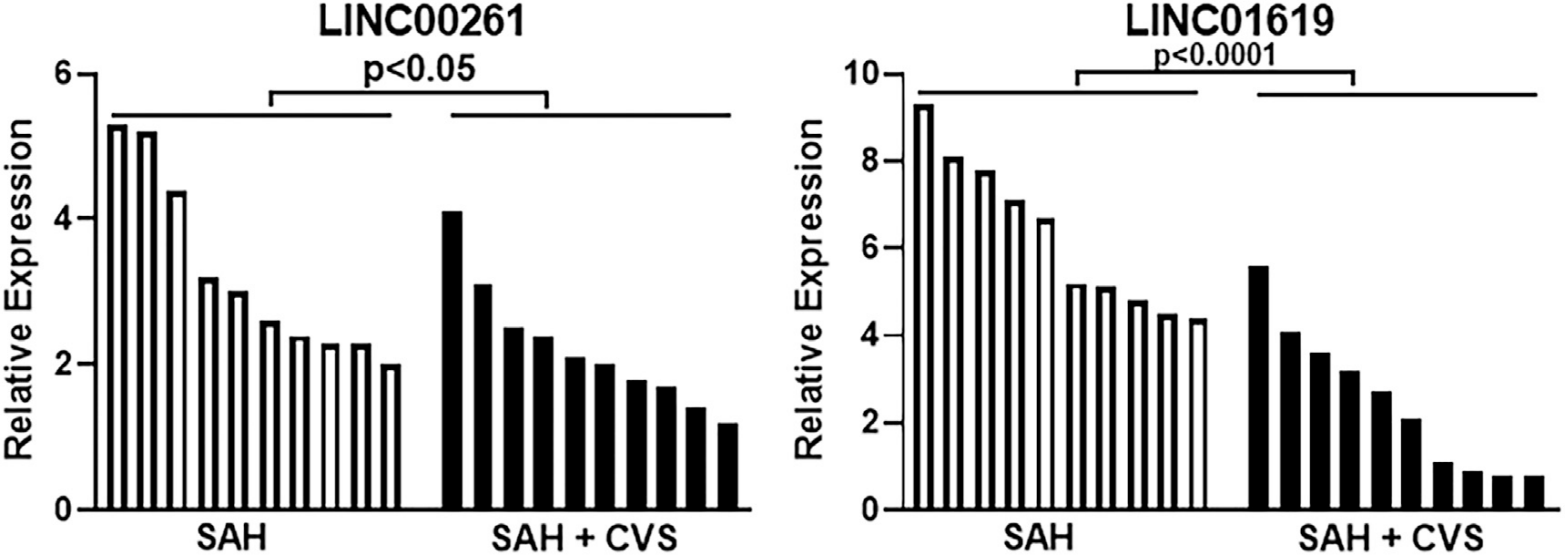
Expression of LINC00261 and LINC01619 in Individual Patient Samples Expression levels of lncRNAs LINC00261 and LINC01619 in individual samples representing patients with SAH (without CVS) and those with SAH and CVS. Values plotted are mean ± 95% CI.

We first looked at expression of upregulated lncRNAs (in SAH + CVS samples, relative to SAH-alone samples) ZFAS1 and MALAT1. As seen in Figure 3, the relative expression of lncRNAs in SAH + CVS samples was higher than the SAH-alone samples, but clearly, MALAT1 expression seemed to be more distinct, as most of the SAH + CVS patients had a higher expression of MALAT1 compared to SAH patients without CVS. Just one patient in the SAH + CVS group had MALAT1 expression lower than that of three SAH-alone patients. The groups were found to be statistically different, as one-way ANOVA yielded a p value of 0.0402 and 0.00003 for ZFAS and MALAT1, respectively. Nest, we also evaluated the expression of lncRNAs LINC00261 and LINC01619, the ones downregulated in SAH with CVS compared to SAH without CVS. Of the two lncRNAs, LINC01619 stood out as the one in which the expression levels were much reduced in SAH patients with CVS (Figure 4). Just one patient in the SAH + CVS group had higher LINC01619 levels than five patients in the SAH-without CVS group. The groups were found to be statistically different, with a p value of 0.0430 and 0.00008 for LINC00261 and LINC01619, respectively.

### Prediction of CVS in SAH Patients Using the lncRNA Signature

**Figure 5:**
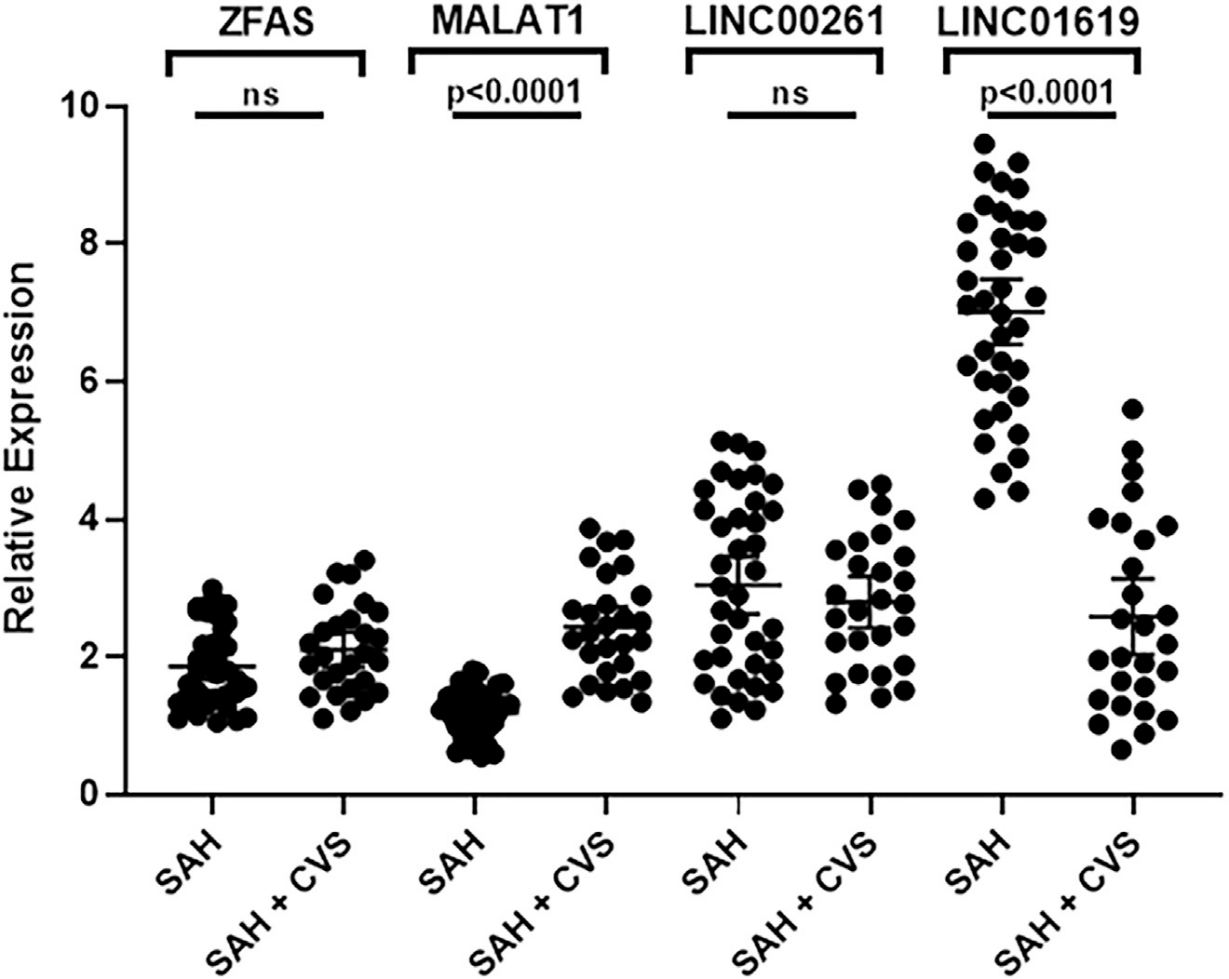
Validation of lncRNA Signature in Archived Samples Expression levels of lncRNAs ZFAS1, MALAT1, LINC00261, and LINC01619 in individual archived samples are shown. 38 samples were from patients without CVS, whereas 27 samples represented patients with SAH and CVS. Values plotted are mean ± 95% CI. ns, nonsignificant.

**Figure 6:**
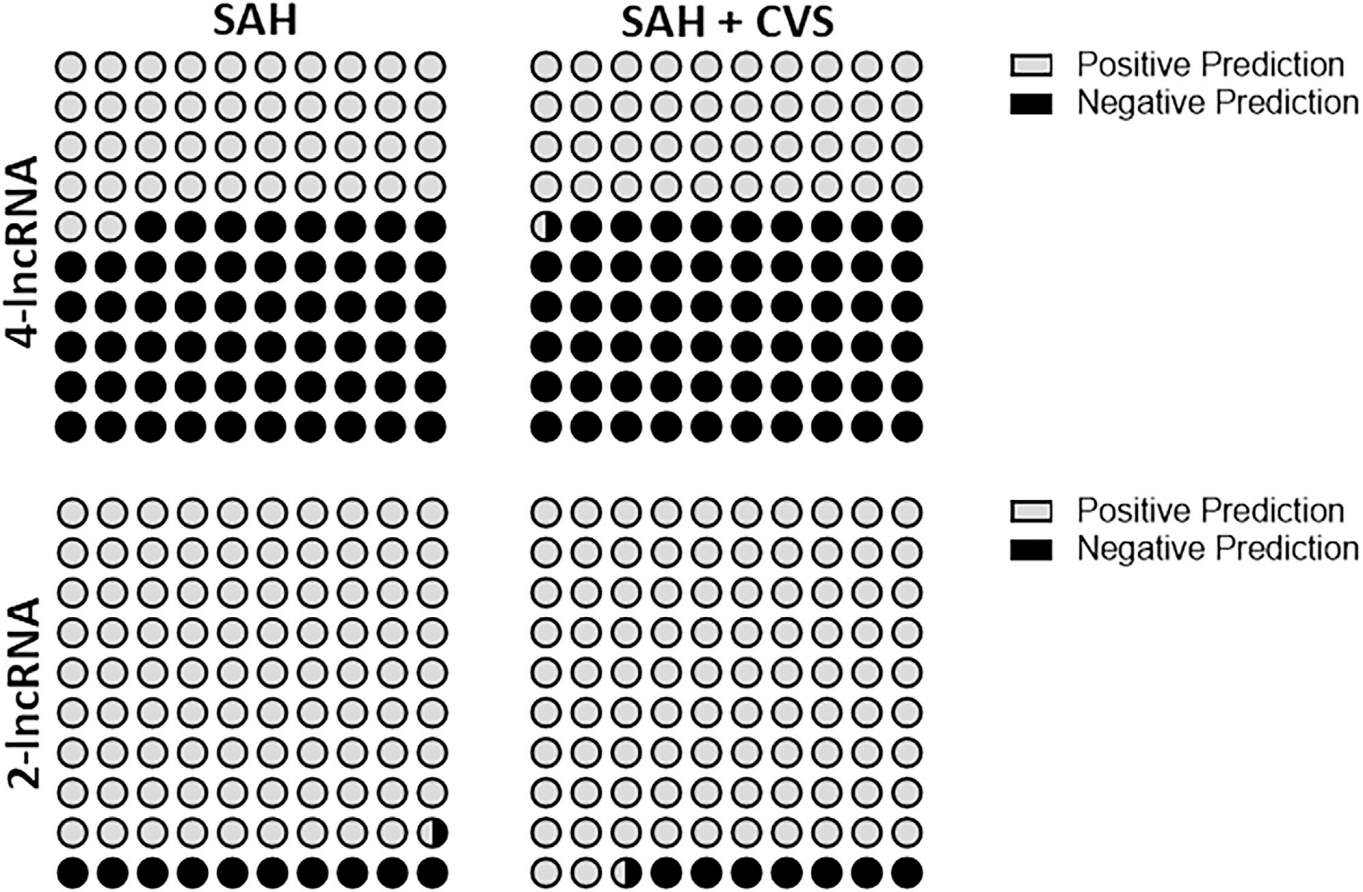
Predictive Power of lncRNA Signatures Predictive power of a 4-lncRNA signature versus 2-lncRNA signature in predicting occurrence of CVS in patients with SAH. 4-lncRNA signature consisted of lncRNAs ZFAS, MALAT1, LINC00261, and LINC01619, whereas the 2-lncRNA signature only considered MALAT1 and LINC01619.

As discussed so far, we found 4 lncRNAs in which the expression levels could potentially help predict SAH. We tested this lncRNA signature to retrospectively predict CVS in patients with SAH. At our institute’s facility, we found 65 cases with a complete history and diagnosis. Of these, 38 samples were from patients without CVS, whereas 27 samples were from patients with CVS. We analyzed the expression levels of four lncRNAs in all of these 65 cases (Figure 5) and then, based on the expression of lncRNAs, predicted whether or not the patient would progress to CVS. When we used the expression of all 4 lncRNAs, the 4-lncRNA signature, we found that the predictions were not very accurate (Figure 6). Less than one-half of the predictions was correct. This was true for both SAH patients without CVS, as well as SAH patients with CVS.

Since two lncRNAs, MALAT1 and LINC01619, stood out as the two lncRNA with more distinct expression patterns in SAH patients with CVS, compared to SAH patients without CVS, we decided to evaluate the predictive power of these two lncRNAs, the 2-lncRNA signature, to predict the occurrence of CVS. As seen in the lower panel of Figure 6, the 2-lncRNA signature predicted occurrence (as well as nonoccurrence) of CVS with more precision (a little less than 90% accuracy for SAH without CVS and more than 90% accuracy for SAH with CVS).

## Discussion

SAH is a type of stroke.^12^ A number of risk factors for SAH have been identified that include primarily elevated blood pressure and alcohol and drug abuse.^4^^,^^13^ About 20% of all strokes are hemorrhagic, with SAH accounting for one-half of them.^14^^,^^15^ Age has also been evaluated as a risk factor, and based on a study comprising 8,144 cases, it was concluded that the elderly population actually has a decreased risk and incidence of aneurysmal SAH.^16^ Interestingly, the analysis found that the incidence rate in females starts decreasing after an age of 65 years, whereas in males, it starts decreasing after an age of 53 years.

The pathophysiology of SAH and the following CVS remains largely unknown.^17^^,^^18^ CVS is a very common event in SAH patients, reported in 70% of aneurysmal SAH patients.^19^ It has a rather predictable course, with delayed onset between 3 and 5 days post-SAH and delayed narrowing of cerebral arteries between days 5 and 14.^19^ A number of treatment modalities are available to either prevent or treat CVS. These include calcium channel blockers (nimodipine, nicardipine, verapamil, and fasudil), magnesium, statins, hormones (erythropoietin and estrogen), phosphodiesterase inhibitors (milrinone, papaverine, and cilostazol), endothelin-1 antagonist (clazosentan), nitric oxide, heparin, and fibrinolysis.^19^ Just the availability of these many treatment options could be misleading, because the prognosis of CVS is rather poor, which has necessitated the need for a better understanding of its onset and etiology.

The use of noncoding RNAs as biomarkers has been increasingly realized in multiple diseases, including a role of miRNAs in predicting or detecting recurrence after surgery.^20^ Of course, there are multiple reports demonstrating their importance as biomarkers and prognostic markers of glioma, the most common tumor affecting the central nervous system.^21^, ^22^, ^23^ More recent research has highlighted the importance of noncoding RNAs in human diseases, even though they were considered junk up until a few years back.^24^ In addition to miRNAs, lncRNAs are also being studied in glioma.^25^^,^^26^

In our present study, we found that a 2-lncRNA signature comprising lncRNAs MALAT1 and LINC01619 is better than a 4-lncRNA signature comprising these two lncRNAs along with lncRNAs ZFAS1 and LINC00261. Clearly, lncRNAs ZFAS1 and LINC00261 were taking away from the predictive power of MALAT1 and LINC01619. This might be because of some overlap in the expression profile of SAH patients without CVS and the SAH patients with CVS, in the context of lncRNAs ZFAS1 and LINC00261. The observation is difficult to explain, and we hope that we unearth some mechanistic details sooner. It is interesting to mention that LINC01619 can actually act as a sponge for miR-27a.^27^ miR-27a is one of the two miRNAs that were proposed by Stylli et al.^9^ to help predict the occurrence of CVS on SAH patients. Also, MALAT1 is predicted to target miR-451a, as per the DIANA tools LncBase Experimental v.2.^28^ miR-451 was also one of the miRNAs shortlisted in the Stylli et al.^9^ study.

### Conclusions

Our results point toward the possible utility of lncRNAs as the predictors of CVS in patients with SAH. miRNA–lncRNA interactions are well documented and may influence the disease progression, but in our study, we did not evaluate the combined predictive power of miRNAs and lncRNAs. Such studies should be a meaningful evaluation moving forward.

## Materials and Methods

### Patient Population

All patients were enrolled at Jilin University Hospitals between January 2018 and December 2018. The study was conducted after approval from the Ethics Committee at the Jilin University (approval number 17/2471). Informed consent was obtained from all patients prior to the collection of samples.

For this study, we recruited 10 patients (6 females and 4 males) with SAH without CVS and 10 patients (6 females and 4 males) with SAH and CVS. As the control groups, we recruited 10 individuals (6 females and 4 males). The mean age of the SAH-without-CVS group was 54.1 years, and the SAH and CVS group was 55.0 years. Mean age of controls was 54.9 years. Characteristics of all controls and patients are provided in Table 1.

**Table 1:** Patient Characteristics

Patient #	Age	Gender	Angiographic Spasm	Admission CT Fisher SAH Grade	Symptomatic Spasm	Symptomatic Spasm Day (Post-SAH)	Ruptured Aneurysm Location
Controls							
C1	56	M	N/A	N/A	N/A	N/A	N/A
C2	51	M	N/A	N/A	N/A	N/A	N/A
C3	66	F	N/A	N/A	N/A	N/A	N/A
C4	39	F	N/A	N/A	N/A	N/A	N/A
C5	48	F	N/A	N/A	N/A	N/A	N/A
C6	72	F	N/A	N/A	N/A	N/A	N/A
C7	60	M	N/A	N/A	N/A	N/A	N/A
C8	49	M	N/A	N/A	N/A	N/A	N/A
C9	53	F	N/A	N/A	N/A	N/A	N/A
C10	55	F	N/A	N/A	N/A	N/A	N/A
SAH (without CVS)							
S1	48	M	no	4	no	N/A	Rt MCA
S2	55	F	no	4	no	N/A	ACoA
S3	68	F	no	4	no	N/A	Lt MCA
S4	38	F	no	4	no	N/A	PCoA
S5	45	M	no	4	no	N/A	Rt MCA
S6	51	F	no	4	no	N/A	Lt pericallosal
S7	56	M	no	4	no	N/A	ACoA
S8	69	F	no	4	no	N/A	Lt MCA
S9	65	M	no	4	no	N/A	Lt SCA
S10	46	F	no	4	no	N/A	basilar tip
SAH + CVS							
SC1	55	F	severe	4	yes	6	Lt pericallosal
SC2	64	F	severe	4	yes	12	Lt MCA
SC3	62	M	mild	4	yes	7	Rt MCA
SC4	69	F	severe	4	yes	6	Rt MCA
SC5	39	M	severe	4	yes	9	basilar tip
SC6	41	F	moderate	4	yes	10	PCoA
SC7	47	F	moderate	4	yes	11	Lt MCA
SC8	54	M	severe	4	yes	14	ACoA
SC9	56	M	mild	4	yes	8	ACoA
SC10	63	F	moderate	4	yes	13	Lt SCA

ACoA, anterior communicating artery; CVS, cerebral vasospasm; MCA, middle cerebral artery; N/A, not applicable; PCoA, posterior communicating artery; SCA, superior cerebral artery; M, male; F, female; Rt, right; Lt, left.

Clinical management of patients was accomplished, as described by Stylli et al.^9^ Briefly, brain computed tomography (CT) scanning or the presence of blood/xanthochromia in the cerebrospinal fluid (CSF) collected via lumbar puncture was employed to diagnose SAH. Symptomatic hydrocephalus was treated with an inserted ventriculostomy catheter and drainage of CSF, as assessed by the in-charge neurosurgeon. All patients were subjected to cerebral digital subtraction angiography (DSA) for the diagnosis of aneurysm location and its morphology. Postsurgery, patients were taken care of at our neurosurgical facility. All patients were subjected to insertion of a central venous catheter. They were administered supplementary fluids to maintain mild hypervolemia and a central venous pressure (CVP) target of greater than 8 cm H_2_O. Patients suspected to develop CVS, but who had a CVP lower than 8 cm H_2_O, were administered fluid bolus (0.9% saline) to restore CVP.

### Specimen Collection and Storage

CSF was collected daily, and, for this study, the period ranged from 1 to 16 days (mean 7.3 days). The duration of drainage was assessed and determined by the in-charge neurosurgeon. The CSF collected for individual patients over several days was all pooled before further analysis. For the control patients, CSF was collected via a lumbar puncture. As selected by Stylli et al.,^9^ these were patients with sudden-onset headache but normal results on brain CT scanning. The lumbar puncture excluded SAH with no other identifiable cause for headache. All of the samples were collected and centrifuged at 2,000 × *g* for 5 min at 4°C to remove contaminating blood cells.^9^ Aliquots were stored in a −80°C freezer.

### RNA Preparation

We used RNAiso reagent (TaKaRA, China) to isolate RNA by following the exact instructions provided by the vendor. Chloroform (1.2 times of starting sample) was mixed to the samples and mixed well by vortexing. After incubation at room temperature for 5 min, centrifugation was done at 12,000 × *g* for 15 min at 4°C. The top layer was transferred into a fresh tube and measured and 0.5 × propanol added, followed by vortexing for even mixing. After an incubation at room temperature for 10 min, centrifugation was done at 12,000 × *g* for 10 min at 4°C to yield the RNA pellet. 75% cold ethanol was used to wash the pellet, followed by centrifugation at 7,500 × *g* for 5 min at 4°C. The RNA pellet was air dried and resuspended in nuclease-free water. Quality of RNA was checked using a NanoDrop 2000 instrument (Thermo Fisher Scientific, China). Only samples with optical density (OD)_260_/OD_280_ >1.8 were considered suitable for analysis. The integrity of RNA was further checked by a Bioanalyzer (Agilent Technologies, Japan) using an RNA 6000 Nano LabChip.

### qRT-PCR

We used primers and detection reagents purchased from QIAGEN (China) to detect lncRNAs in our patient samples. Only RNase-free water was used throughout the assays. We first used the RT^2^ First Strand Kit (QIAGEN, China) for the synthesis of cDNA. This kit ensures complete elimination of genomic DNA. The starting amount of RNA was 1 μg to which 2 μL of genomic DNA elimination mix was added and mixed by pipetting, followed by incubation for 5 min at 42°C and then immediate transfer to ice for 1 min. Reverse transcription mix, consisting of 5× buffer and Reverse Transcriptase, was prepared exactly as suggested and added to the tube containing RNA. This was incubated for 15 min at 42°C and then the reaction stopped by transfer for 5 min to 95°C.

The RT^2^ lncRNA qPCR assay (QIAGEN, China) was used for the detection of lncRNAs. The product from the RT^2^ First Strand step was mixed with RT^2^ SYBR Green Master Mix (QIAGEN, China) and run on an ABI 7500 RT-PCR system (Applied Biosystems) with the following PCR cycle conditions: 1 cycle—10 min/95°C, followed by 40 cycles consisting of two steps—15 s/95°C and 1 min/60°C.

### Validation in Archived Samples

The lncRNA signature obtained in the present study was used to predict CVS in archived CSF samples. A total of 65 samples were identified that had complete clinical history available. 38 (21 females and 17 males) samples represented patients with no CVS, whereas 27 (15 females and 12 males) samples were from patients with CVS. A double-blind evaluation was conducted wherein deidentified samples were subjected to lncRNA detection, and a prediction was made based on their lncRNA signature. Detailed documentation was then assessed to verify if the lncRNA signature could help identify SAH patients who developed CVS.

### Statistical Considerations

All data were reviewed and analyzed by a trained statistician who was blinded to the identity of individual patients as well as groups. To evaluate if 2 datasets were significantly different, a p value was calculated using Student’s t test or one-way ANOVA, assuming equal variables and 2-tailed distribution. Prior to the statistical tests, datasets were log transformed to ensure normal distribution. When statistical tests were performed on a large number of variables, to eliminate false discovery rate, obtained p values were converted into *q* values. For the predictive analysis, we used the log-linear model described by Ouyang et al.^29^ to describe transcript abundance. Only the p values ≤0.05 were considered to represent statistically significant analyses.

## Author Contributions

C.-Y.P., M.T., L.-L.Z., and L.-Y.W. conducted experiments and collected data. C.-Y.P., M.T., D.T., L.-Y.W., and Y.-J.S. analyzed results and created figures. D.T. and Y.-J.S. performed statistical analysis. C.-Y.P. and M.T. prepared the first draft. All authors corrected and proofread the manuscript. Y.-F.C. supervised the study.

## Conflicts of Interest

The authors declare no competing interests.
